# Phylogenomic analysis of the bowfin (*Amia calva*) reveals unrecognized species diversity in a living fossil lineage

**DOI:** 10.1038/s41598-022-20875-4

**Published:** 2022-10-03

**Authors:** Jeremy J. Wright, Spencer A. Bruce, Daniel A. Sinopoli, Jay R. Palumbo, Donald J. Stewart

**Affiliations:** 1 Research & Collections, New York State Museum, 3140 Cultural Education Center, Albany, NY USA; 2 Department of Information Technology Services, University at Albany–State University of New York, Albany, NY USA; 3 Department of Biological Sciences, Museum of Natural Sciences, Louisiana State University, Baton Rouge, LA USA; 4 Department of Environmental Science & Ecology, State University of New York at Brockport, Brockport, NY USA; 5 Department of Environmental Biology, State University of New York College of Environmental Science and Forestry, Syracuse, NY USA

**Keywords:** Evolution, Molecular evolution, Phylogenetics, Population genetics, Speciation, Taxonomy

## Abstract

The Bowfin (*Amia calva*), as currently recognized, represents the sole living member of the family Amiidae, which dates back to approximately 150 Ma. Prior to 1896, 13 species of extant Bowfins had been described, but these were all placed into a single species with no rationale or analysis given. This situation has persisted until the present day, with little attention given to re-evaluation of those previously described nominal forms. Here, we present a phylogenomic analysis based on over 21,000 single nucleotide polymorphisms (SNPs) from 94 individuals that unambiguously demonstrates the presence of at least two independent evolutionary lineages within extant *Amia* populations that merit species-level standing, as well as the possibility of two more. These findings not only expand the recognizable species diversity in an iconic, ancient lineage, but also demonstrate the utility of such methods in addressing previously intractable questions of molecular systematics and phylogeography in slowly evolving groups of ancient fishes.

## Introduction

“Living fossils”, or relictual taxa that are recognizable from the fossil record and maintain many aspects of ancestral phenotypes, are found throughout the tree of life and offer invaluable insights into the evolutionary development of modern organisms^1^. Fishes that fall under this categorization have particularly been of recent interest for their potential to provide insight into the genomic architecture and mechanisms underlying the evolution of teleosts and land-dwelling tetrapods^2–6^. These groups of fishes are generally characterized by lower species diversity, slower rates of molecular evolution in protein-coding genes and, in the case of species in the Infraclass Holostei (Bowfin and Gars), smaller genome sizes relative to teleosts^2–7^. In holosteans, these genomic characteristics have been attributed, at least in part, to their divergence prior to a whole-genome duplication (WGD) at the base of the teleost radiation, which has been hypothesized to have facilitated teleost diversification through the adaptive evolution of newly available paralogs of existing genes^4,6,8–10^, although differing viewpoints and caveats have begun to gain some support^11–14^. The same aspects of their genomes that have caused holostean fishes to become emerging model systems in studies of vertebrate genome evolution also, however, have the potential to complicate molecular phylogenetic examinations of these taxa, with multiple loci generally needed to fully resolve intra- and interspecific relationships^15,16^.

**Figure 1:**
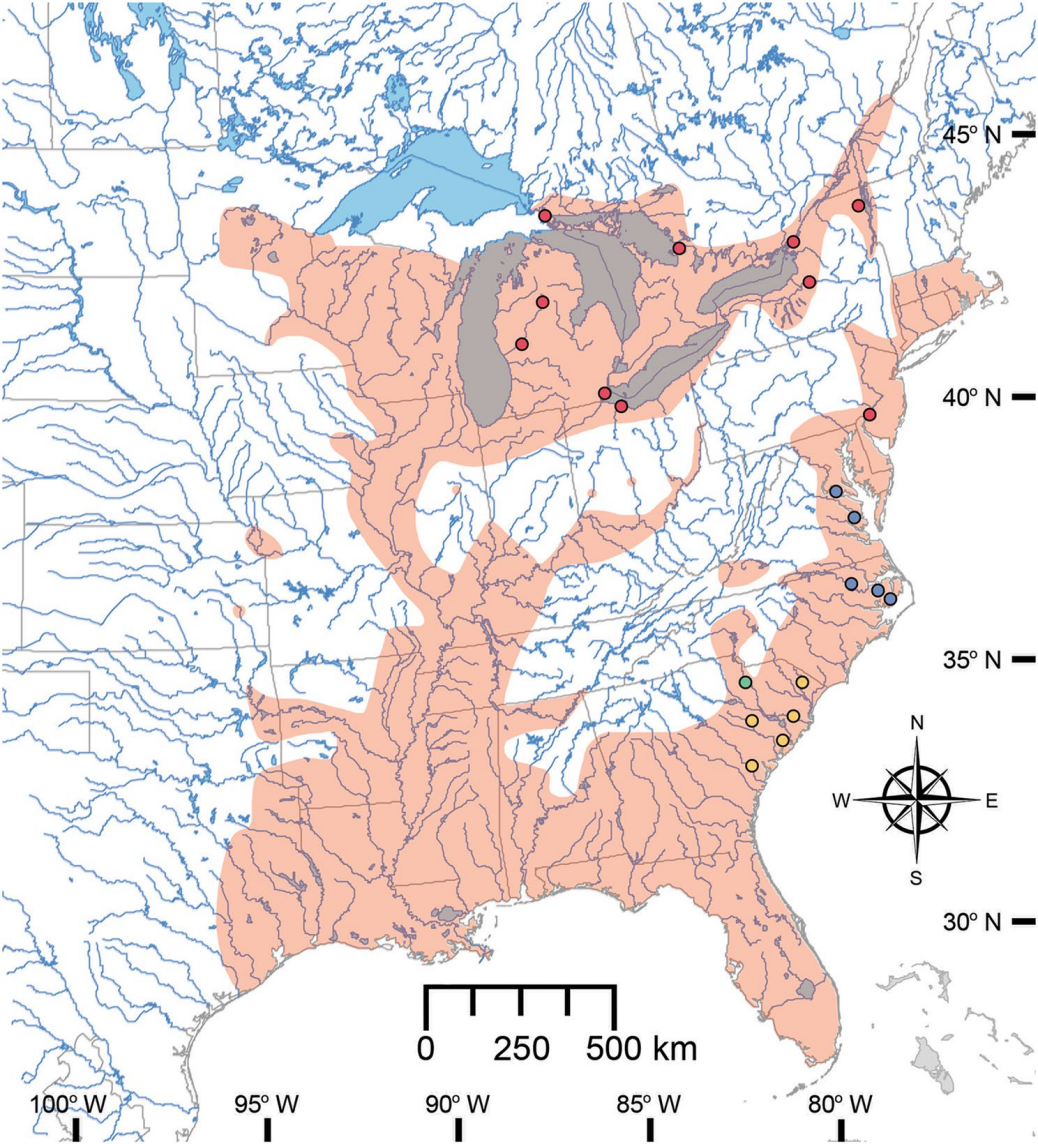
Currently recognized distribution of *Amia calva* (red shading). Dots represent sampling locations, with colors corresponding to population clusters shown in Fig. 2a. Range is redrawn from Page and Burr^17^, using a modified version of a map of North American lakes and rivers provided by the Commission for Environmental Cooperation (http://www.cec.org/north-american-environmental-atlas/lakes-and-rivers-2009/).

Bowfins are relatively large (to 109 cm total length^17^), predatory fishes that are found from southern Canada to southern Florida, U.S.A., and westward to lowland areas of the Mississippi River basin and Gulf Coast drainages from southern Texas to western Florida, with non-native populations in New England (Fig. 1). *Amia calva* Linnaeus 1766 is currently considered to represent the only living member of its family (Amiidae) and order (Amiiformes), with fossil *Amia* spp. known from as early as the late Paleocene (≈ 55 Ma)^18,19^. Prior to 1871, however, 13 species of extant *Amia* had been recognized and named (Fig. S1 and Table S1). In 1896, Jordan and Evermann placed all 12 additional nominal Bowfin taxa in the synonymy of *A. calva*, although they offered neither analysis nor rationale for doing so^20^. In the 125 years since, the species-level taxonomy and phylogenetics of the genus *Amia* has remained highly static, with descriptions of two new fossil taxa^18^, possible regional variation^21^, and demonstration of population genetic structure in the southern United States^22^, but there has been no systematic, critical examination of Jordan and Evermann’s hypothesis of monotypy. It follows that there have also been very few examinations of Bowfins focusing on processes that may have influenced the diversification of these fishes. These are puzzling oversights (but a testament to the power of conventional wisdom), as the Bowfin is a cornerstone in the study of comparative vertebrate anatomy and evolution, and both living holostean lineages have received masterful examinations of their skeletal and external morphology and the phylogenetic relationships supported by those characters^18,23^.

Here, we present a phylogenetic analysis of restriction-site associated DNA sequencing (RAD-Seq) data derived from 94 individual Bowfin specimens from several populations in the Laurentian Great Lakes basin, as well as multiple populations from Atlantic Coastal drainages (Fig. 1 and Table S2). Data such as these have successfully been used in molecular phylogenetic analyses of fishes^24–27^ and other organisms^28–32^ where accurate resolution of relationships was previously hampered by rapid diversification and short branch lengths, uninformative markers, or relationships that were otherwise difficult to resolve. The slow rate of genetic evolution in Bowfin presents a similar problem, making these data and analyses an obvious choice for the detection of possibly unrecognized species diversity in this group. When viewed in conjunction with morphological data, our results provide unambiguous support for the recognition of at least one additional living species of *Amia* and reveal additional interesting population structure in Atlantic Coastal populations. Examining the variation in our data with respect to the Bowfin genome allows for even deeper levels of interpretation, identifying possible targets of adaptive selection that may be continuing to drive extant Bowfin evolution and diversification.

## Results

### Assembly, mapping and SNP calling

We compared different k-mer sizes for assembly of the trimmed and error-corrected paired-end reads and found that k = 96 produced the fewest scaffolds and highest N50 score. The resulting k = 96 de novo reference assembly created for read mapping resulted in 135,802 contigs with a total length of 777,297,260 bp and an N50 score of 10,688, which was better than expected for an assembly built entirely of RAD-Seq reads. Raw reads mapped to the reference assembly resulted in 21,145 SNPs after filtering calls with a minor allele frequency ≥ 0.10, removing indels, and excluding sites with more than 50% missing data.

### Phylogenetic and population genetic analysis

**Figure 2:**
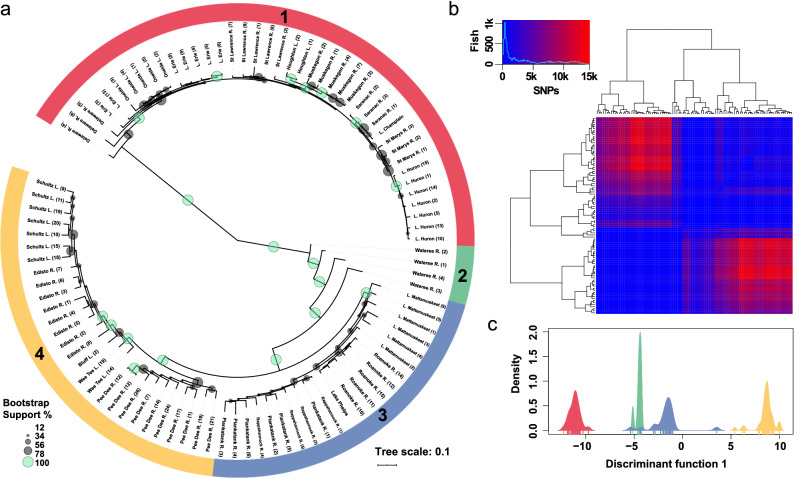
(**a**) Phylogenetic population structure of sampled Bowfin (*Amia* spp.) based on 21,145 SNPs. Branches with 100% bootstrap support are colored in green and population genomic analysis carried out with hierBAPS detected 4 primary population clusters, demarcated with the colored, numbered rings (for rectangular phylogram, see Supplementary Fig. 2). (**b**) Heatmap depicting pairwise SNP differences, histogram and color key indicate distribution of SNPs across samples. (**c**) DAPC analysis results based on discriminant function 1, density plot is colored according to population designation in (**a**).

Phylogenetic analysis of the SNP dataset revealed a deep split between Laurentian Great Lakes (plus Delaware River) and Mid-Atlantic Coastal populations, with 100% bootstrap support (Fig. 2a and Fig. S2). Bootstrap values varied widely across the dataset, however, ranging anywhere from 12 to 100%. Pairwise SNP distances ranged from 61 to 1452 SNPs (mean = 3897) across all samples examined (Fig. 2b). Relationships within the Great Lakes clade were characterized by very short branches and low bootstrap support, paralleling mitochondrial patterns of postglacial recolonization in another holostean fish, the Spotted Gar (*Lepisosteus oculatus*)^16^. The Delaware River individuals were most closely related to specimens from Lake Erie, indicating that this population may represent an introduction rather than a native occurrence, as has been suggested elsewhere^33,34^. Well-supported phylogenetic substructure was observed within the Coastal Plain populations, with clades representing Middle Atlantic systems, the Little Pee Dee River (South Carolina), and other South Carolina lowland populations all supported by 100% bootstrap values. Again, very little additional well-resolved phylogenetic structure was seen within these subclades. An interesting pattern was observed in samples from the Wateree River Basin (i.e., Catawba R. in Piedmont habitat, above the Fall Line), in which all four samples were recovered as paraphyletic with respect to other Coastal Plain individuals, again with 100% bootstrap support. The inclusion of additional samples in future analyses may clarify or resolve this pattern (but see below).

Hierarchical cluster analysis detected four clusters (C1–C4) within the dataset after converging at a local optimum. These clusters are largely reflective of the phylogenetic structure described above, with the exception that both South Carolina Coastal Plain subclades (Pee Dee River and southward) were included in C4. As indicated above, bootstrap support for the nodes subtending these four clusters was estimated at 100%. As might be expected from the phylogenetic results, C1 is separated from C2–C4 by a comparatively large number of SNPs, ranging from 761 to 14,252 SNPs (mean = 7043), while the number of SNPs separating C2–C4 from the rest of the group ranged anywhere from 422 to 8624 SNPs, 143–14,252 SNPs, and 143–13,550 SNPs, respectively (mean = 3961, 3617, and 5602). Results of a discriminant analysis of principal components largely mirrored those of the cluster analysis, inferring four population groups and strong differentiation between C1 and C4 (Supplementary Fig. S3). A density plot exhibiting the number of samples across discriminant function 1, colored according to their hierBAPS designation, is shown in Fig. 2c.

**Figure 3:**
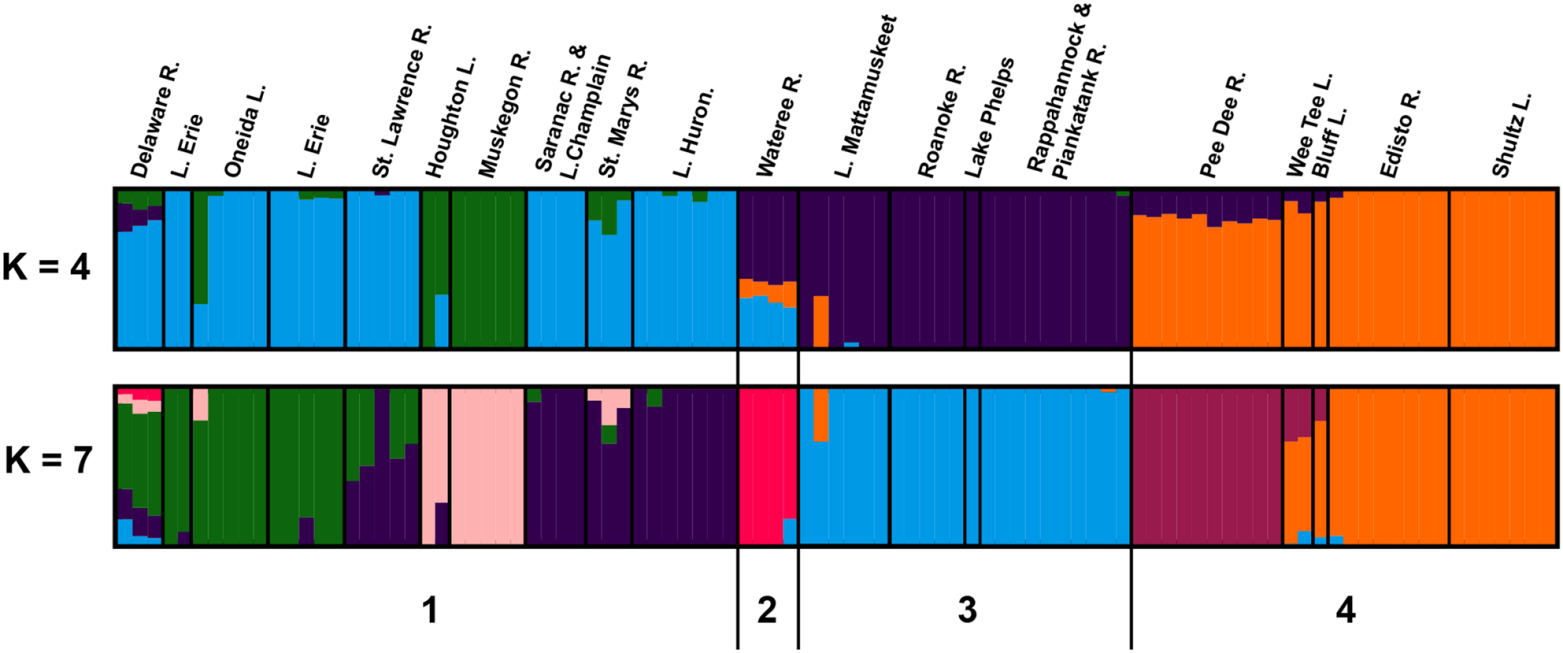
Results of admixture analysis for K = 4 (CV error = 0.25) and K = 7 (CV error = 0.27). Samples are ordered according to the tree in Fig. 2a and labels at the bottom correspond to population clustering as determined using hierBAPs.

The results of an admixture analysis yielded two K-values with nearly equivalent levels of cross-validation error [K = 4 (CV error = 0.25) and K = 7 (CV error = 0.27); Fig. 3 and Supplementary File 1]. Both estimates showed results that are consistent with phylogenetic and clustering analyses, but also revealed patterns that merit the collection of additional specimens and data. Delaware River individuals again showed a much greater affinity with Great Lakes populations than with Atlantic Coastal Plain populations (> 80% for both K = 4 and K = 7; Fig. 3; Supplementary Files 2, 3). The ancestry of our Wateree River individuals remained ambiguous; at K = 4, admixture with Great Lakes and Atlantic Coastal Plain populations was indicated, while at K = 7, this population showed genetic structure that was quite distinctive (Fig. 3; Supplementary Files 2, 3). We do not, at present, have a definitive explanation for this pattern, but it is clearly worthy of further investigations that incorporate data from additional specimens (see below). Houghton Lake and Muskegon River individuals also showed distinct genetic structure, though small amounts of admixture between these populations and other Great Lakes populations were observed (Fig. 3; Supplementary Files 2, 3). Additional, finer-level investigations of Bowfin population genetics in the Great Lakes region are needed to identify the presence and geographical extent of this structure, as well as any additional, potentially informative, genetic diversity in these populations.

### Adaptation and gene ontology

**Figure 4:**
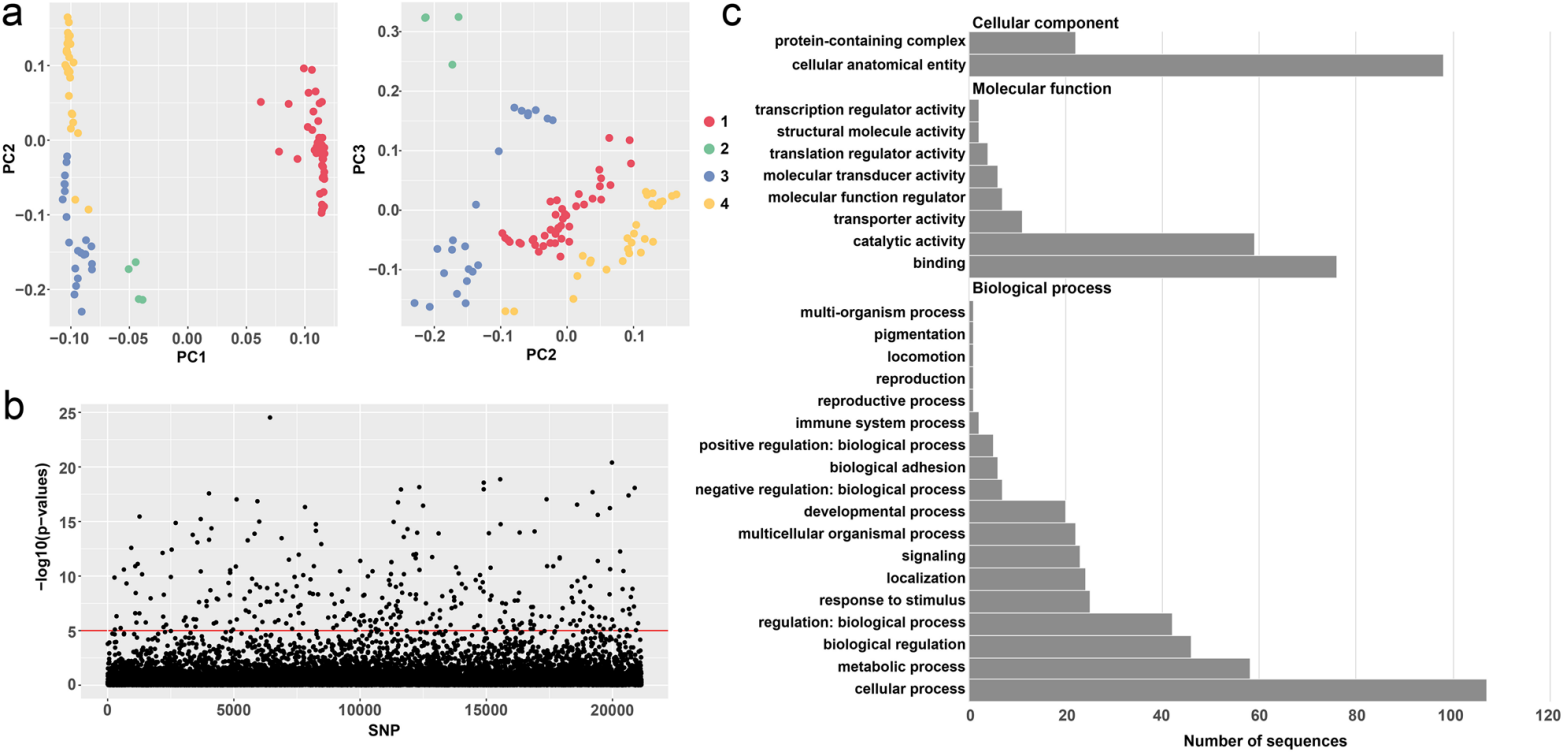
(**a**) PC projections of PCs 1–3, dots are colored according to the population designation determined with hierBAPS. (**b**) Manhattan plot showing all SNPs, significance as determined by the Bonferroni method is demarcated in red. (**c**) Gene ontology analysis, number of sequences related to the three hierarchal categories and their sub-classes are shown.

To assess the optimal K used to carry out the pcadapt analysis we examined the percentage of variance explained by 20 principal components in addition to projections comparing PCs 1 through 6 (Figs. 4 and S4). The PC projections, along with the scree plot showed strong support for K = 2 and thus was used for downstream analysis. Significant *P*-values determined using the Bonferroni method identified 289 candidate SNPs for selection across our mapped reads (Fig. 4b). Contigs containing potential adaptive loci were then analyzed using OmicsBox. Results of the NCBI basic local alignment searches, Gene Ontology Annotation database matches and Interpro database matches are available upon request to the corresponding author. Gene classifications related to gene annotation for contigs containing potential adaptive loci are shown in Fig. 4c. Most candidate loci were found on sequences associated with biological processes (n = 392), while a lesser number were associated with molecular function (n = 167) and cellular components (n = 120). The majority of sequences were associated with adaptation related to cellular processes (n = 107), followed closely by cellular anatomical activity (n = 98). Molecular functions such as catalytic activity (n = 59) and binding (n = 76) demonstrated an intermediate number of sequences associated with selection, as well as biological processes such as metabolic processes and several processes related to regulation (n = 42 and 46, respectively). All other sequences were present in numbers < 25.

## Discussion

**Figure 5:**
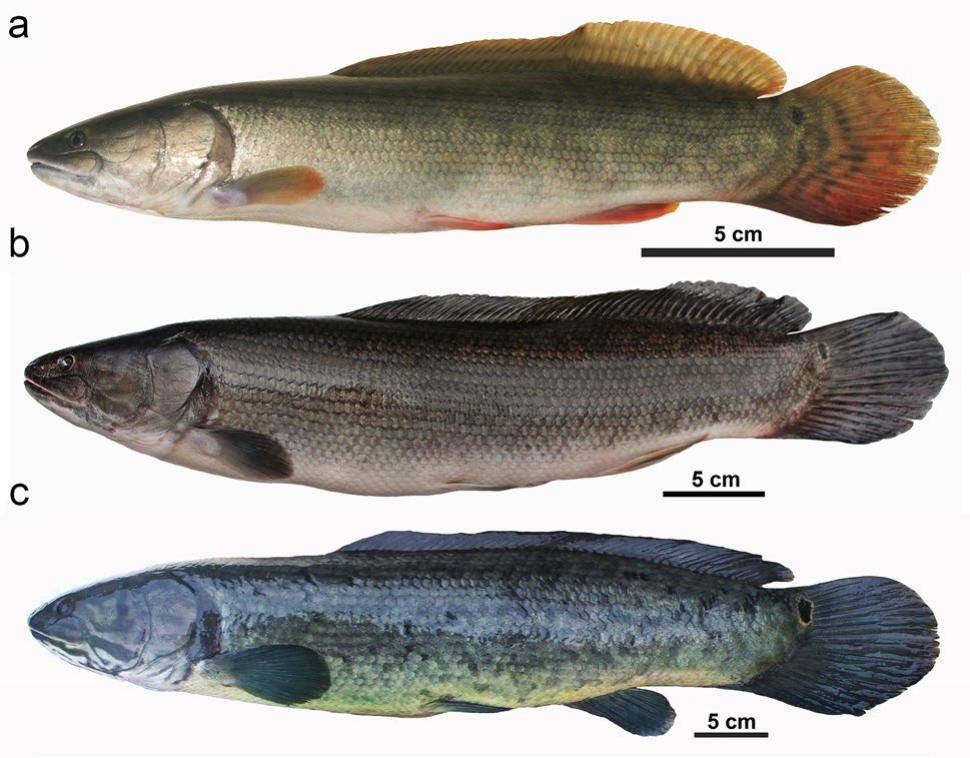
(**a**) *Amia calva* from the lower Savannah River, SC, showing live colors. (**b**) Freshly preserved *A. calva* from Schultz Lake, SC, which drains into the south side of Charleston Harbor (the reported type locality of *A. calva*). (**c**) Putative specimen of *A. ocellicauda* (potential neotype; Royal Ontario Museum 99787) from Georgian Bay, ON, showing contrasting color pattern to (**a**) and (**b**); photos by D.J.S.

The presence of genetic diversity within Bowfins has been known for several decades^22^ but, to our knowledge, has not been the focus of systematic or taxonomic investigations in that time. We have presented unambiguous molecular evidence for the presence of at least two living *Amia* species with more likely to exist, perhaps even among the populations examined here. This analysis primarily focuses on two of five biogeographic regions of the U.S.A. with native Bowfin populations, Great Lakes and Central Appalachian^35,36^; additional comparative sequencing and analysis of Bowfins from other regions are currently underway. This conclusion is further supported by several meristic and morphometric characters that clearly distinguish the populations examined in our present study (Fig. S5, Tables S3–S6). Geographic provenance and taxonomic priority indicate that our Great Lakes specimens are representative of *Amia ocellicauda* Todd 1836 (Fig. 5) pending a formal redescription of that taxon (DJS, DAS, JJW, *In preparation*).

Our observed phylogenomic patterns for Bowfins are consistent with ichthyofaunal observations concerning the Central Appalachian Province, which extends from the Susquehanna River basin in the north to the Edisto River basin in South Carolina^35^. For example, the northern areas of the Mid-Atlantic coast (e.g., James and Roanoke drainages) have eight endemic fish species, and the southern area (e.g., Santee and Pee Dee drainages) have six endemics. Results also indicate the range of *A. calva* extends southward at least to the Savannah River basin, which is in the Southeastern Province. An early study of mtDNA patterns among Bowfins in the southern U.S. found that the genotype for the Cooper River, SC, population was distributed westward only to the Apalachicola River basin on the Georgia-Alabama border^22^. So, it could be that the range of *A. calva* extends from the Pee Dee basin south and westward to the Apalachicola basin and southward into the Florida Peninsula. This is a hypothesis that should be tested with more samples from Georgia, Florida, and Alabama.

Coastal Plain aquatic habitats in South Carolina are (or were) dominated by baldcypress (*Taxodium distichum*) swamps, which appear to be favored by *A. calva*. Lower and Upper Coastal Plain habitats have substrates that are primarily fine sediments of Pleistocene and Pliocene ages, respectively (South Carolina Department of Natural Resources Geological Survey; https://scdnr.maps.arcgis.com/apps/Viewer/index.html?appid=735411a2f5714f28a424422296f77bb1). In contrast, Piedmont habitats have more rocky substrates, are mostly of older geologic ages, and historically never had baldcypress forests. Upper reaches of the Wateree basin appear to be the only Piedmont habitat along the Mid-Atlantic Coast where a native Bowfin population extends far upriver. We suspect that the Wateree population could be a local endemic species; the inclusion of additional specimens in future analyses is necessary to clarify the matter.

Given the wide range of latitudes and environmental conditions in which Bowfin populations are found, it should not, perhaps, be surprising that a majority of the loci in which we detected evidence of adaptive evolution are related to potentially temperature-sensitive factors such as cellular and subcellular structures, binding, and catalytic activity. Thermal tolerances, responses, and optima are complex, polygenic, epigenetic traits that have significant impacts at all levels of biological organization and life history and, among other abiotically-influenced factors, have been implicated to play a role in local adaptation in a number of geographically widespread organisms^e.g.,^^37–40^. An examination of the precise nature and fitness impacts of potentially adaptive changes in our various Bowfin candidate loci is beyond the scope of the present study, but overall patterns suggest that physiological adaptations driven by environmental factors may have played a significant role in the formation and maintenance of species diversity in *Amia*. What, if any, contributions these genetic and physiological adaptations have made to regional morphological differences observed in Bowfin populations similarly remains to be seen.

Much has been made of the depauperate nature of extant holostean biodiversity, with the assumption being that most of the species and genetic diversity of these families have been lost to past extinctions, including the three recognized species of fossil *Amia*^4,6,18,23^. While this is no doubt true in an overarching sense, our study offers a counter perspective to the idea that only a single living amiid species remains, with living Bowfins retaining considerable genomic and taxonomic variation that should be explored to further facilitate understanding of vertebrate evolution. In addition, it has been noted that many fragmentary *Amia* fossil materials exist that are inadequately diagnosed, and that “*Amia* provide a remarkable case study on nomenclatural problems resulting from poorly preserved paleospecies.”^18^. Observations of regional variation in the morphology of living *Amia* such as those indicated above have the potential to inform taxonomic treatments of existing and future fossil materials.

In rejecting the hypothesis of extant Bowfin monotypy, we have been conservative in our estimate of the species diversity represented by our samples^41^. Under a unified species concept in which species are recognized as separately evolving metapopulation lineages^42,43^ up to four species, corresponding to the four major lineages and population clusters recovered in our analyses, might reasonably be recognized from the individuals sampled. There are, however, limits to the conclusions that can be drawn from our analyses, largely due to small sample sizes from some (meta) populations^41^. This is most clearly seen in the paraphyly of our Wateree River Basin individuals although similar patterns, albeit with much lower bootstrap values, were recovered for Delaware River (New Jersey) and Lake Mattamuskeet (North Carolina) populations (Fig. 2a). Although there are no concrete guidelines regarding the number of individual samples required to confidently delimit species using RAD-Seq data^44^, the inclusion of additional sequence data from these populations clearly has the potential to resolve and/or help to explain these relationships. The clear separation of dozens of individuals from the Great Lakes and similar numbers of individuals from close proximity to the Coastal Plain type locality of *Amia calva* leaves little doubt, however, that at least two species of extant *Amia* exist. This conclusion is further supported by additional ecological and morphological information and data, which fulfill the criteria of some traditional species concepts and further support our recognition of multiple *Amia* species under the unified species concept^43^.

The detection of unrecognized taxonomic diversity in *Amia* likely has future ramifications for conservation efforts, as Bowfin are not currently the subject of regulation throughout much of their geographical range and are often considered a nuisance species by recreational anglers^36^. In addition to persecution by anglers, Bowfin are the target of a burgeoning caviar industry and regional populations, some of which may represent geographically restricted species, have the potential to suffer significant negative impacts to recruitment and standing genetic diversity due to overexploitation. Until such time as the full scope of Bowfin species and population-level diversity is understood, as well as the potential impact of threats (caviar and recreational fishing, habitat loss, invasive species, etc.) to that diversity, a ‘precautionary principle’ such as those advocated by the IUCN and other entities^45–49^ might reasonably be considered to protect currently unrecognized Bowfin diversity.

## Materials and methods

### Specimen acquisition and sequencing

All methods have been reported in accordance with ARRIVE guidelines. Cornell University and Virginia Tech University Institutional Animal Care and Use Committee (IACUC) approval was obtained for collection and euthanization procedures performed by representatives of those institutions. In the case of state and provincial governmental conservation agencies where IACUC approval was not required for the collection and preservation of fishes (see Acknowledgements), all methods were performed in accordance with guidelines recommended by the American Fisheries Society^50^. Bowfin specimens were collected using a combination of boat and backpack electrofishing or by trap nets, and fishes were euthanized by placement on ice. Pelvic fin clips were taken from iced or frozen specimens as soon as feasible and were stored in 95% ethanol at − 80 °C until DNA extraction. Whole specimens were retained, fixed in 10% formalin, and then transferred to 70% ethanol prior to morphological examination and accession to museum collections (see Table S2 for detailed locality data). Dried whole skeletons from most sample areas were prepared (with ‘Ridewood’ cranial dissections and cleaned with dermestid beetles) for ongoing morphological analyses.

Genomic DNA was extracted using a Qiagen DNeasy Blood and Tissue kit (Qiagen, Valencia, CA, USA) according to manufacturer’s instructions, with the addition of a 10-min. incubation with 4.0 µL of RNAse A at 56 °C (10 mg/mL; Thermo Fisher Scientific, Waltham, Massachusetts, USA). DNA was quantified using an Invitrogen Qubit 4 Fluorometer (Thermo Fisher Scientific, Waltham, Massachusetts, USA) and subsamples were diluted to approximately 10–20 ng/µL for use in library construction and sequencing.

Library construction and sequencing were conducted by SNPsaurus, LLC. NextRAD genotyping-by-sequencing libraries were produced as in Rusello et al.^51^. Genomic DNA was first fragmented with Nextera DNA Flex reagent (Illumina, Inc), which also ligates short adapter sequences to the ends of the fragments. The Nextera reaction was scaled for fragmenting 30 ng of genomic DNA, although 60 ng of genomic DNA was used for input to compensate for the amount of degraded DNA in the samples and to increase fragment sizes. Fragmented DNA was then amplified for 27 cycles at 74°, with one of the primers matching the adapter and extending 10 nucleotides into the genomic DNA with the selective sequence GTGTAGAGCC. Thus, only fragments starting with a sequence that can be hybridized by the selective sequence of the primer will be efficiently amplified. The nextRAD libraries were sequenced on a HiSeq 4000 with one lane of 150 bp reads (University of Oregon).

### Genome reference assembly

We created a de novo reference assembly by collecting 10 million reads in total, evenly from the samples. Raw reads were filtered and trimmed with fastp v0.20^52^. To improve contiguity for the final assembly, we used a Cross-Species Scaffolding pipeline to construct mate-pair libraries in silico^53^, employing the filtered and trimmed reads, and using the spotted gar (*Lepisosteus oculatus*) genome (NCBI assembly: GCF_000242695.1). We then extended the length of the filtered and trimmed reads by overlapping paired-end reads from fragment libraries that were sufficiently short using the program FLASH v1.2.11^54^. The program Clumpify v38.90 (from the BBtools package) was then used to remove identical read pairs^55^ and read pairs that mapped to the Bowfin mitochondrial genome were also removed using Bowtie2 v2.2.9^56^. Kmer counting and error correcting of the sequencing reads was carried out with the program musket v4.1.2^57^. We assembled the genome using the trimmed and error‐corrected paired‐end reads and single-end reads with ABySS v2.2.3^58^. To determine the optimal *k*‐mer length, we repeated the assembly using *k* = 88–128 in 8‐bp increments. All scaffolding steps were performed using the trimmed mate‐pair reads in ABySS, and only scaffolds longer than 500 bp were retained.

### Mapping and SNP calling

We used fastp to remove adaptor contamination and trim leading and trailing bases from each read with a phred-scaled quality score (Q) < 20. Additionally, we applied a four-base sliding window to the trailing end and removed additional bases if the average Q across the window was < 20. Finally, we removed all reads shorter than 50 bp and with a mean Q across the entire read < 30. We mapped the trimmed sequence reads to the reference genome using BWA v0.7.12^59^. We also removed duplicate reads and only kept unambiguously mapped and properly paired reads with a mapping quality (MQ) ≥ 20 in SAMtools v1.3^60^. SNP calling was carried out using the ref_map.pl program from the Stacks pipeline v2.53^61^, which runs each of the Stacks components individually using the default parameters. Resulting SNP calls were then processed with VCFtools v0.1.17^62^ to filter SNP calls with a minor allele frequency ≥ 0.10, remove indels, and exclude sites with more than 50% missing data.

### Phylogenetic analysis and population structure

We inferred phylogenetic relationships using the maximum likelihood (ML) method carried out with RAxML v8.2.12^63^ on the resulting SNP dataset. Non-parametric bootstrapping was implemented with 200 replicates, applying the GTR + Γ model of nucleotide evolution (GTRGAMMA) to build a phylogenetic tree. To compliment the ML analysis described above, we also applied a population genomic approach to identify distinct clusters across the SNP dataset. For this analysis, we employed hierBAPS^64,65^, which provides a method for hierarchically clustering DNA sequence data to reveal nested population structure. One level of molecular variation was fitted to the data, and the analysis was run until it converged at a local optimum. For visualization, the phylogenetic tree was mid-point rooted and arranged in decreasing node order with FigTree v1.4.4^66^. Results of the RAxML and hierBAPs analyses were then combined and visualized using iTOL version 4.3^67^. We also used the program pairsnp (https://github.com/gtonkinhill/pairsnp) to create a pairwise SNP distance matrix and visualized the results using the R package ggplot2 v3.3.2^68^. We additionally carried out a discriminant analysis of principal components (DAPC), a multivariate method designed to identify and describe clusters of genetically related individuals with the R package adegenet v2.1.3^69^. Finally, we performed an admixture analysis on all samples with parameters ‘-j4 -cv -C 0.1’ in ADMIXTURE^70^. We tested the number of ancestral populations between K = 1 and K = 10 and applied cross-validation (CV), which identified the optimal values of K = 4 (CV error = 0.25) and K = 7 (CV error = 0.27) shown in Fig. 3.

### Adaptation and related gene ontology

To identify functional differences related to adaptation, we used pcadapt v4.3.3^71^, which performs genome scans for selection based on individual genotypes employing Principal Component Analysis (PCA). We first assessed the percentage of variance explained by 20 principal components in the form of a scree plot in addition to projections comparing PCs 1 through 6 to determine the optimal number of principal components (K) to retain. Subsequent analysis was then performed assuming K = 2 for the whole data set. Candidate genes for selection were identified based on significant *P*-values determined using the Bonferroni method to correct for multiple comparisons. We then extracted contigs from the Bowfin assembly containing SNPs that were significant candidates for selection. The resulting contigs were then scanned and masked using RepeatMasker v4.1.1^72^ employing the Dfam database to avoid non-specific gene hits. To determine gene ontology related to sequences where adaptation was identified we used OmicsBox (https://www.biobam.com/omicsbox/)^73^. OmicsBox provides a suite of functions for the NGS data analysis of genomes, transcriptomes, and metagenomes. Contigs were first blasted using the NCBI basic local alignment search tool^74^, employing the non-redundant protein sequences database, using the Actinopterygii taxonomy filter. The resulting blast hits were then mapped to the Gene Ontology Annotation database^75^ to identify matches. Annotation was then carried out using the most reliable gene ontology terms, considering the gene ontology hierarchy, sequences similarities, and the abundance and quality of the source annotation.

## Supplementary Information

Supplementary Information 1.

Supplementary Information 2.

Supplementary Information 3.

Supplementary Information 4.

## Data Availability

All code related to the genome assembly and read mapping can be found at the following link: https://github.com/spencer411/Bowfin_code. Supplementary File 1 contains cross-validation error values for K = 1 to K = 10 ancestral populations. Supplementary Files 2 and 3 contain admixture estimates for each individual at K = 4 and K = 7 populations, respectively. Raw sequencing reads are available from NCBI under BioProject ID PRJNA875639 (see Supplementary Table 2 for individual accession numbers). The datasets generated and/or analyzed during the current study are available in the Dryad repository (https://doi.org/10.5061/dryad.pzgmsbcq5).
