# Supplementary material for: Phylogenomic analysis of the bowfin (Amia calva) reveals unrecognized species diversity in a living fossil lineage
Source: Sci Rep. 2022 Oct 3;12:16514. doi: 10.1038/s41598-022-20875-4 (PMC9529906; doi:10.1038/s41598-022-20875-4)
Supplement: Supplementary file 4 — Supplementary Information 4. [file 41598_2022_20875_MOESM4_ESM.docx]

**Supplementary Information**

**Phylogenomic Analysis of the Bowfin (*Amia calva*) Reveals Unrecognized Species Diversity in a Living Fossil Lineage**

Jeremy J. Wright^1†^*, Spencer A. Bruce^†2^, Daniel A. Sinopoli^3^, Jay R. Palumbo^4^, Donald J. Stewart^5^

† Authors contributed equally

* Corresponding author

Jeremy Wright Jeremy.Wright@nysed.gov

1 Research & Collections, New York State Museum, 3140 Cultural Education Center, Albany, New York, USA

2 Department of Information Technology Services, University at Albany – State University of New York, Albany, New York, USA

3 Department of Biological Sciences, Museum of Natural Sciences, Louisiana State University, Baton Rouge, Louisiana, USA

4 Department of Environmental Science & Ecology, State University of New York at Brockport, Brockport, New York, USA

5 Department of Environmental Biology, State University of New York College of Environmental Science and Forestry, Syracuse, New York, USA

**Supplementary Text**

**Morphological Data Collection and Analyses.** As a full taxonomic treatment is beyond the scope of this study, we have included a subset of morphological data and analyses of our Bowfin specimens with geographic proximity to the type localities of *Amia calva* (South Carolina Coastal Plains) and *A. ocellicauda* (Georgian Bay, ON). Each fish was assigned a unique identification number and photographed to record live or fresh color and pigmentation patterns. A tissue sample taken from the right pelvic fin of each fish was preserved in 95% ethanol for genetic analyses; sex was recorded when possible. To prevent internal decay and preserve body shape, specimens were injected with full-strength formalin (37% formaldehyde) along the core of the body and then laid out in a straight, natural position for at least 40 min. They were then submerged in a bath of 10% formalin solution for 4-5 days to finalize preservation. Finally, formalin was removed by soaking in water changes for several days, and specimens were then transferred to 70% ethanol for long-term archival.

Protocols for morphological and meristic sampling were developed^1,2^, refined^3^, and applied to the whole-fish vouchers supporting the genetic analyses that are the focus of this paper. Data were taken from left, dorsal, and ventral aspects of each specimen based on various morphological landmarks. Morphometric characters (N=38, see Supplementary Table 1 for definitions) were measured with digital calipers and meristic characters (N=8, Supplementary Table 2) were counted for each specimen. Morphometric measurements 100 mm or greater were measured to the nearest 1.0 mm, while those under 100 mm were measured to the nearest 0.1 mm. A 60 cm digital caliper was used for measurements 30 cm or greater, and a 30 cm digital caliper was used for measurements under 30 cm.

Some morphometric measurements and meristic counts used in this study differed from those presented in a previous monograph on Bowfins^4^ because those previously defined characters were intended to be useful for taxonomic comparisons among extant and fossil fishes. Therefore, in certain cases, those measurements involved only bony landmarks; in contrast, some measurements for this study involved fleshy features that might not be evident in a fossil. Differences were as follows:

1) Standard length (SL) was defined as from tip of snout to most posterior extent of hypurals; herein, SL was measured posteriorly to point where lateral line intersects caudal-fin base (determined as point of caudal flexure, so that would be closely similar).

2) Caudal peduncle length was defined as from posterior end of anal-fin base to most posterior extent of the hypurals (as with SL, according to the text description, but an illustration indicates measuring to dorsal origin of caudal-fin base). In this study, it was measured to posterior end of lateral line where it meets caudal-fin base, as with SL, and thus, in agreement with the prior text definition.

3) Head length was measured from tip of snout to posterior bony margin of operculum; in this study, it was measured to most posterior extent of fleshy opercular flap.

4) Postorbital length was measured from posterior margin of orbit to posterior end of 4th postinfraorbital bone (comparable to ‘fourth infraorbital length’ herein); in this study, postorbital length was measured to posterior-most fleshy margin of opercular flap, as with head length.

5) Scale rows below lateral line were counted from origin of anal fin antero-dorsally to lateral line but not including lateral-line scale; herein, that count began at pelvic-fin origin.

6) Count for scale rows above lateral line were from origin of dorsal fin postero-ventrally to but not including lateral-line scale; herein, that count started where previous transverse scale count ended at lateral line and extended postero-dorsally to dorsal-fin base (excluding lateral-line scale and small scales along dorsal fin base).

7) Fin-ray counts for dorsal and anal fins distinguished unsegmented, rudimentary rays at origins of those fins from segmented rays; herein, all rays of dorsal and anal fins were counted without distinguishing the rudimentary rays from segmented rays. Typically, there are 1-2 rudimentary rays at origin of each fin.

For analyses, measurements were converted to ratios relative to standard length (measured from anterior tip of upper jaw to end of lateral line on caudal-fin base), except posterior nostril diameter (converted to ratio of interorbital width). Morphometric ratios were subjected to Principal Component Analysis (PCA) and Analysis of Covariance (ANCOVA) using the statistical package PAST, MAC Ver. 4.10^5^. In addition, eight meristic characters (counts of repeated structures) were taken from each specimen. Again, we used PAST to perform nonparametric Mann-Whitney U-tests to evaluate any between-population median (rank) differences for meristic counts.

Figure S1: Bowfin distribution (shaded area) with type localities of the 13 nominal species of extant *Amia* as follows: 1 = *Amia calva*; 2 = *A. ocellicauda*; 3 = *A. occidentalis*; 4 = *A. marmorata*; 5 = *A. ornata*; 6 = *A. viridis*; 7 = *A. canina*; 8 = *A. lentiginosa*; 9 = *A. subcoerulea*; 10 = *A. cinerea*; 11 = *A. reticulata*; 12 = *A. thompsonii*; 13 = *A. piquotii*. Numbers reflect date/page priorities based on original descriptions (see Supplementary Table 1 and citations therein). Range is redrawn from Page & Burr^6^, using a modified version of a map of North American lakes and rivers provided by the Commission for Environmental Cooperation (http://www.cec.org/north-american-environmental-atlas/lakes-and-rivers-2009/)


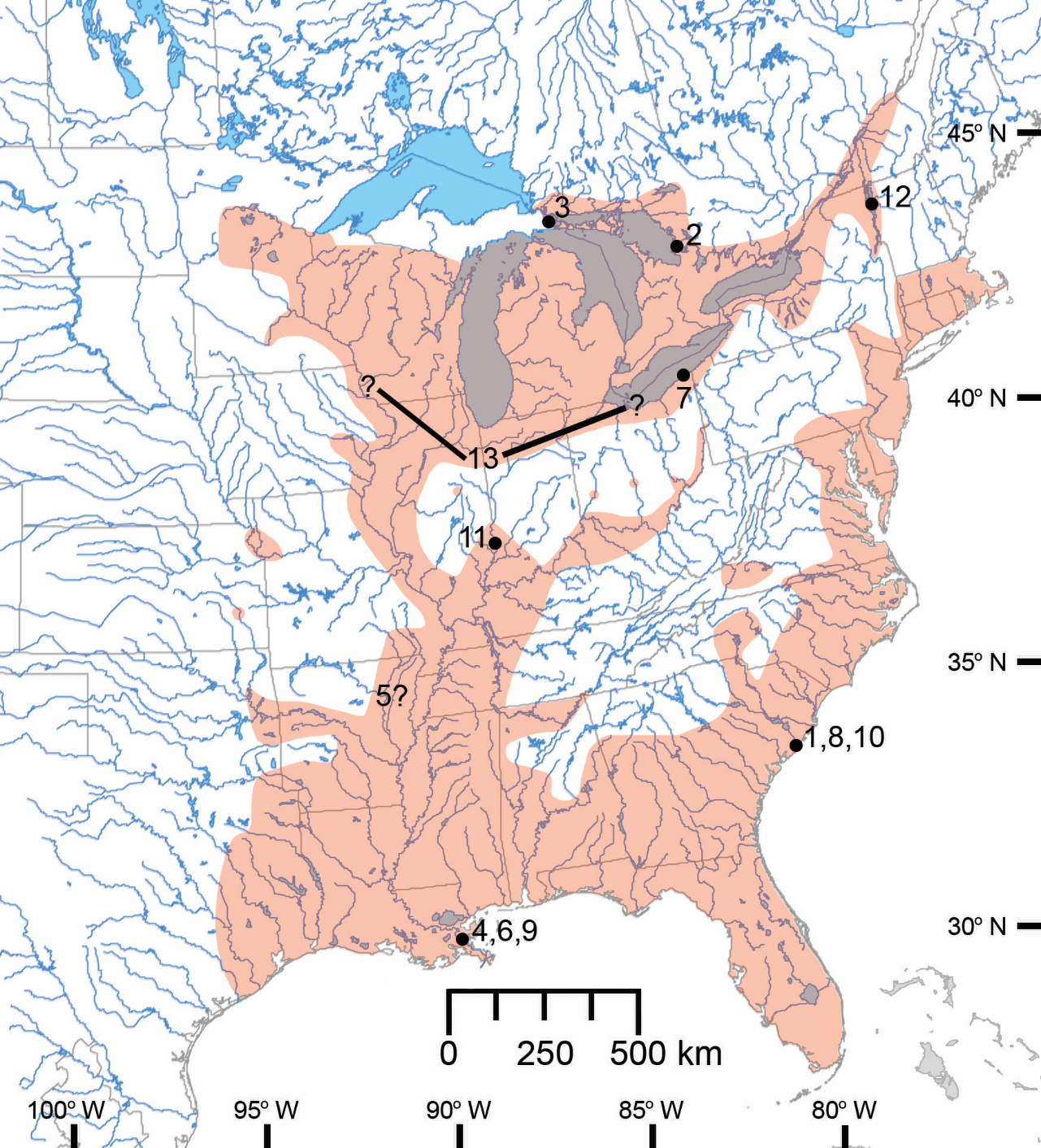


**Figure S2:** Phylogram from Fig. 2A in rectangular format, to aid in interpretation of branch lengths.


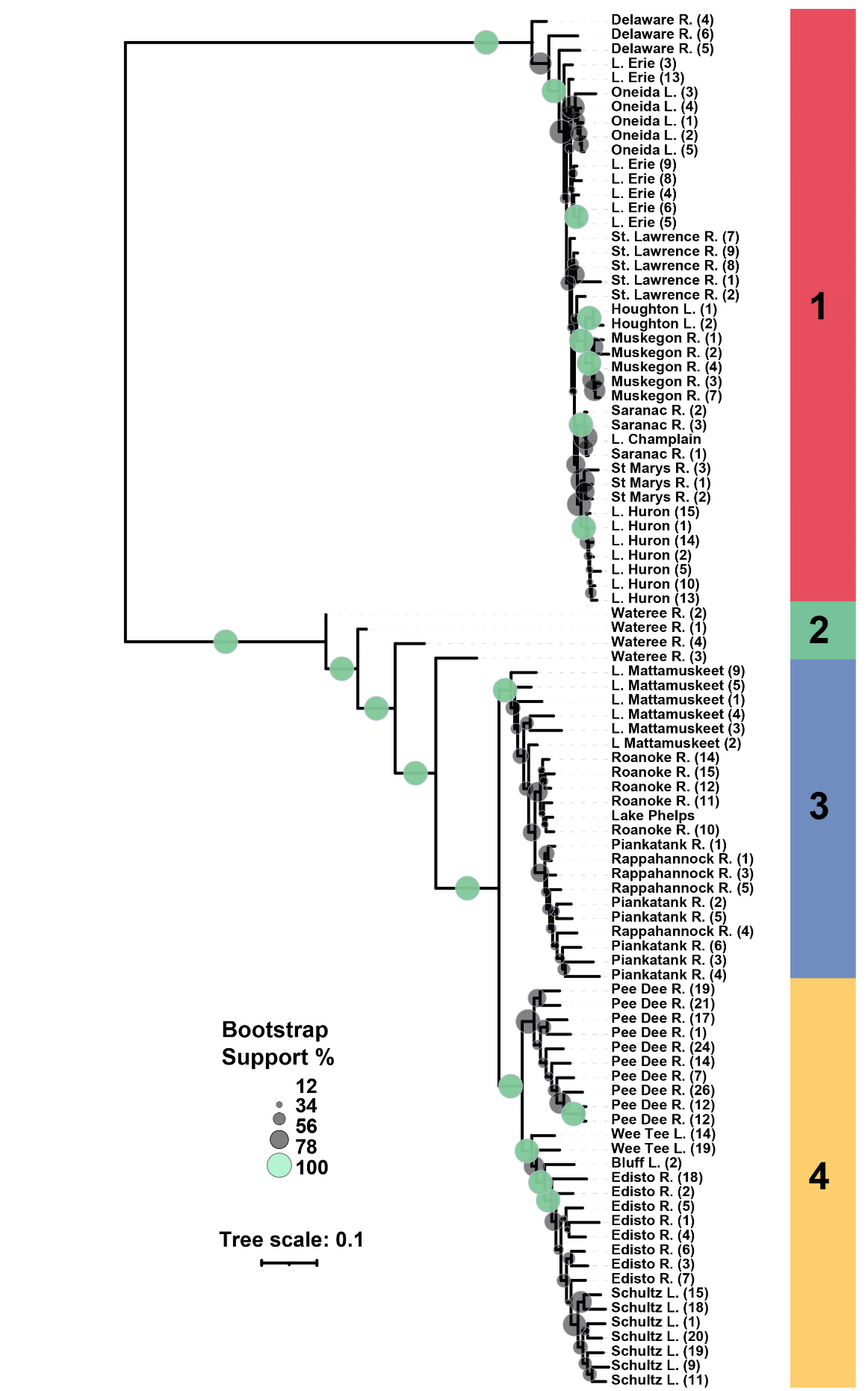


**Figure S3:** Bayesian information criterion (BIC) values and inferred clusters for all samples, produced using the R package adegenet v2.1.3. A BIC value of ~425 represents the “elbow” of the curve, suggesting 4 populations. Rows correspond to hierBAPs designations, while columns correspond to inferred groups (”inf”) designated using the “find.clusters” function. The size of each black square is representative of the number of individual fish across the inferred clusters.


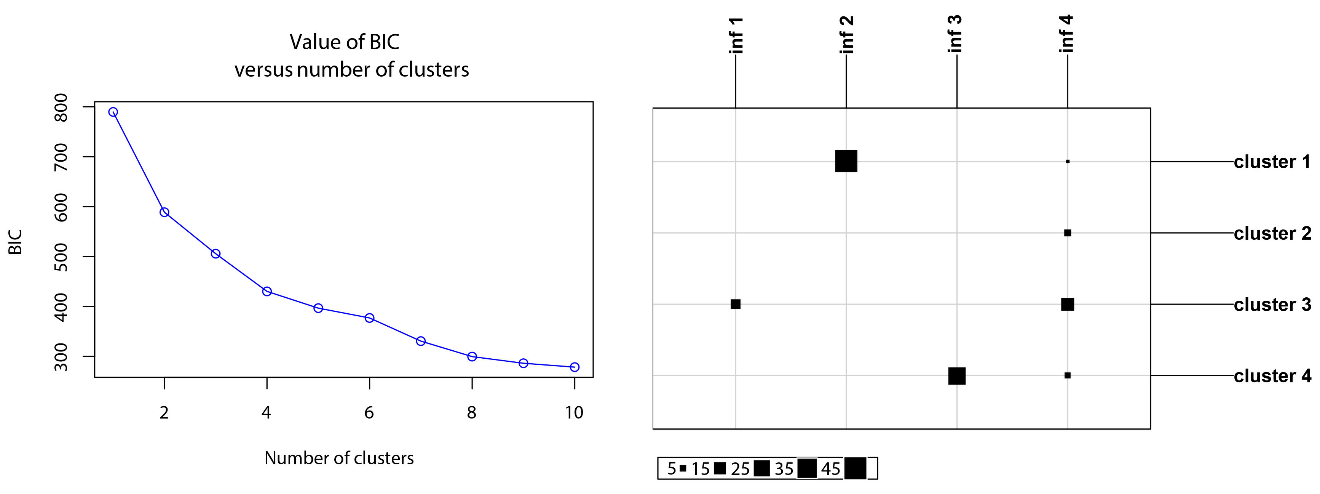


Figure S4: The percentage of variance explained by 20 principal components in the form of a scree plot and projections comparing PCs 1 through 6 produced by pcadapt. Color keys for the projections correspond to the 4 groups identified by hierBAPS (1, 2, 3, and 4; also see Fig. 3 of main text).


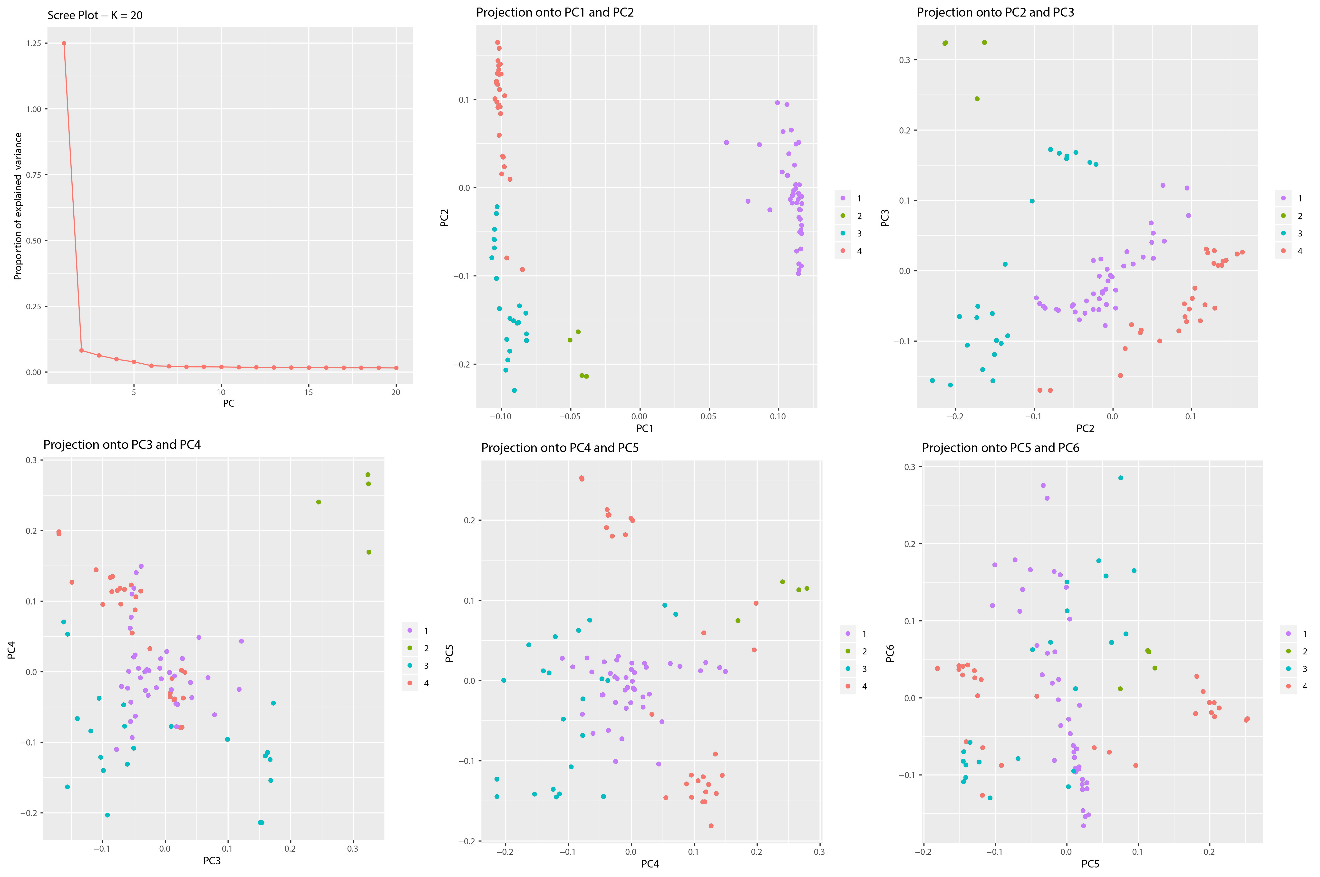


**Figure S5:** Principal components analysis comparing Bowfins from Lake Huron (N=14, blue diamonds = *Amia ocellicauda* topotypes; prospective Neotype indicated by asterisk) with those from coastal plains of South Carolina (red dots, N=41, including likely topotypes of *A. calva*; Little Pee Dee River = green squares, N=10). Based on variance-covariance matrix of 38 morphometric characters (expressed as proportion of standard length, and posterior nostril diameter as proportion of interorbital width); plotted on eigenvalue scale (using PAST for MAC, ver. 4.10, downloaded Mar. 2022^5^)


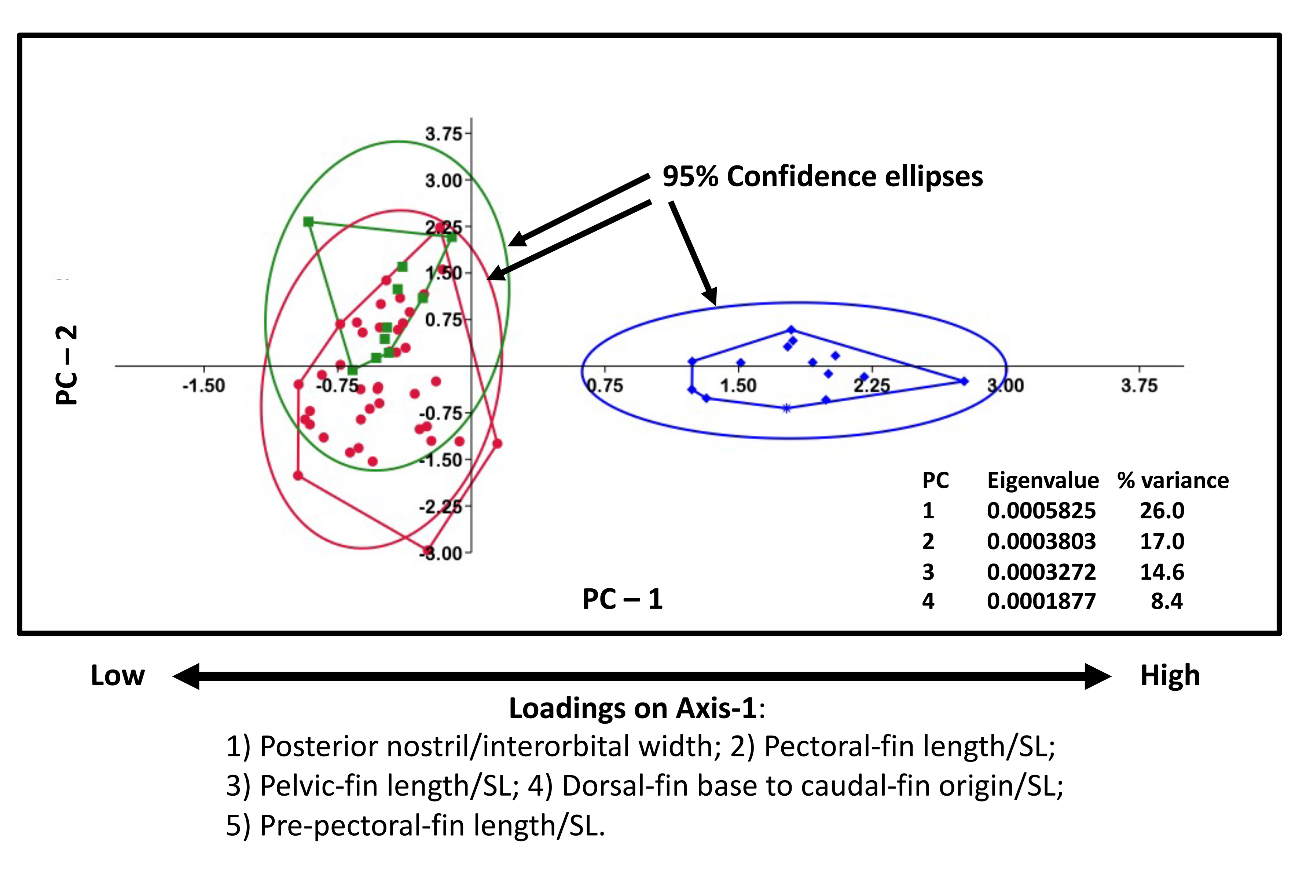


Table S1: Type specimens of the 13 nominal species of extant *Amia* (see Fig. S1 for type localities). The three nominal species of fossil *Amia* were all described after these extant taxa. Abbreviations: CLNH = Cabinet of the Lyceum of Natural History, New York City (Collections burned in 1866); MNHN = Muséum National D’Histoire Naturelle, Paris; ‘King’s Cabinet’ predates use of catalogue numbers in Paris; LSL = Linnean Society of London; P=photos, M=Morphology, X=x-ray. All available type specimens of extant *Amia* are catalogued at LSL and MNHN; they were studied by DJS as a critical foundation for resolving taxonomy of the genus.

| **Species** | **Ref.** | **Type Status** | **Specimen Status** | **Mus.** | **Mus. Cat. #** | **Present Data** |
| --- | --- | --- | --- | --- | --- | --- |
| *A. calva* | 7 | Holotype, Text | Dry-mounted | LSL | 128 | P, M |
| *A. ocellicauda* | 8 | Holotype, Text | Lost in transit | N/A | N/A | N/A |
| *A. occidentalis* | 9 | Holotype, Text, Illust. | Dry-mounted,  Burned, 1866 | CLNH | None | N/A |
| *A. marmorata* | 10 | Holotype, Text, Illust. | Alcohol | MNHN | 1305 | P, M, X |
| *A. ornata* | 10 | Holotype, Text | Alcohol (Juv.) | MNHN | 1568 | P, M, X |
| *A. viridis* | 10 | Text only | Not preserved | N/A | N/A | N/A |
| *A. canina* | 10 | Holotype, Text, Illust. | Lost or not preserved? | N/A | N/A | N/A |
| *A. lentiginosa* | 10 | Holotype, Text, 2 Illust. | Lost in Museum | MNHN | ‘King’s Cabinet’ | N/A |
| *A. subcoerulea* | 10,11 | Holotype/Lectotype, Text | Alcohol | MNHN | 5769 | P, M, X |
| *A. subcoerulea* | 10,11 | Paralectotype-no status | Alcohol | MNHN | 5768 | P, M, X |
| *A. cinerea* | 10 | Holotype, Text | Alcohol | MNHN | 5770 | P, M, X |
| *A. reticulata* | 10 | Holotype, Text | Dry-mounted | MNHN | 5808 | P, M, X |
| *A. thompsonii* | 12 | 2 Syntypes, Text | Alcohol | MNHN | 5766 | P, M, X |
| *A. piquotii* | 12 | 2 Syntypes, Text | Dry-mounted | MNHN | 5813, 5814 | P, M, X |

Table S2: Detailed collection locality information for the Bowfin specimens included in this study. Sample labels correspond to those in Fig. 2a. Quotation marks indicate identical information to the preceding entry. Institutional codes: NYSM = New York State Museum; ROM = Royal Ontario Museum; UMMZ = University of Michigan Museum of Zoology. All but one of the genetic samples reported herein are supported by freshly collected, formalin-preserved vouchers listed below; most also are complemented by photo vouchers.

| **Sample** | **Locality** | **Cat. #** | **Lat.** | **Long.** | **NCBI #** |
| --- | --- | --- | --- | --- | --- |
|  |  |  |  |  |  |
| Delaware R. (4) | Little Mantua Creek, 1 mi. ENE Paulsboro, Gloucester Co., NJ | NYSM 91510 | 39.838558 | -75.2133 | SAMN30626224 |
| Delaware R. (5) | “ | “ | “ | “ | SAMN30626225 |
| Delaware R. (6) | “ | “ | “ | “ | SAMN30626226 |
| L. Erie (3) | East Harbor State Park, 7.5 mi. ENE Port Clinton, Erie Co., OH | NYSM 91511 | 41.54464 | -82.801345 | SAMN30626240 |
| L. Erie (4) | “ | “ | “ | “ | SAMN30626241 |
| L. Erie (13) | “ | “ | “ | “ | SAMN30626239 |
| L. Erie (5) | Sterling State Park, edge of Lake Erie, directly E of Monroe, Monroe Co., MI | NYSM 91512 | 41.921333 | -83.335907 | SAMN30626242 |
| L. Erie (6) | “ | “ | “ | “ | SAMN30626243 |
| L. Erie (8) | “ | “ | “ | “ | SAMN30626244 |
| L. Erie (9) | “ | “ |  | “ | SAMN30626245 |
| Oneida L. (1) | Cornell Biological Field Station, Shackleton Point, Madison Co., NY | NYSM 91513 | 43.173486 | -75.929708 | SAMN30626264 |
| Oneida L. (2) | “ | “ | “ | “ | SAMN30626265 |
| Oneida L. (3) | “ | “ | “ | “ | SAMN30626266 |
| Oneida L. (4) | “ | “ | “ | “ | SAMN30626267 |
| Oneida L. (5) | “ | “ | “ | “ | SAMN30626268 |
| St Lawrence R. (1) | French Creek Bay, Clayton, Jefferson Co., NY | NYSM 91514 | 44.234167 | -76.091389 | SAMN30626303 |
| St Lawrence R. (2) | “ | “ | “ | “ | SAMN30626304 |
| St Lawrence R. (7) | Saint Lawrence River, 5 km NE of Clayton, Jefferson Co., NY | NYSM 91521 | 44.26708 | -76.02869 | SAMN30626305 |
| St Lawrence R. (8) | “ | “ | “ | “ | SAMN30626306 |
| St Lawrence R. (9) | “ | “ | “ | “ | SAMN30626307 |
| Houghton L. (1) | Houghton Lake Flats, off old U.S. Hwy 27, Roscommon Co., MI | UMMZ uncat. | 44.363292 | -84.800247 | SAMN30626235 |
| Houghton L. (2) | “ | “ | “ | “ | SAMN30626236 |
| Muskegon R. (1) | Brooks Lake, 1.5 mi. SE Newaygo, Newaygo Co., MI | “ | 43.395683 | -85.749711 | SAMN30626259 |
| Muskegon R. (2) | “ | “ | “ | “ | SAMN30626260 |
| Muskegon R. (3) | “ | “ | “ | “ | SAMN30626261 |
| Muskegon R. (4) | “ | “ | “ | “ | SAMN30626262 |
| Muskegon R. (7) | “ | “ | “ | “ | SAMN30626263 |
| Saranac R. (1) | Saranac River at Rte 22 crossing, Plattsburgh, Clinton Co., NY | NYSM 69539 | 44.686 | -73.451 | SAMN30626293 |
| Saranac R. (2) | “ | “ | “ | “ | SAMN30626294 |
| Saranac R. (3) | “ | “ | “ | “ | SAMN30626295 |
| Lake Champlain | Wilcox Dock, Plattsburg, Clinton Co., NY | Tissue only | 44.708664 | -73.445981 | SAMN30626238 |
| St Marys R. (1) | Neebish Island, Chippewa Co., MI | UMMZ uncat. | 46.325081 | -84.209214 | SAMN30626308 |
|  |  |  |  |  |  |
| **Sample #** | **Locality** | **Cat. #** | **Lat.** | **Long.** | **NCBI #** |
|  |  |  |  |  |  |
| St Marys R. (2) | Little Lake George, 1 mi. E Sault Ste Marie, Chippewa Co., MI | “ | 46.533717 | -84.194233 | SAMN30626309 |
| St Marys R. (3) | “ | “ | “ | “ | SAMN30626310 |
| L. Huron (1) | Severn Sound, Georgian Bay, Ontario, Canada | ROM 99785 | 44.8691 | -79.8455 | SAMN30626250 |
| L. Huron (10) | “ | “ | “ | “ | SAMN30626246 |
| L. Huron (15) | “ | “ | “ | “ | SAMN30626249 |
| L. Huron (2) | “ | ROM 99784 | “ | “ | SAMN30626251 |
| L. Huron (5) | “ | ROM 99786 | 44.7878 | -79.7396 | SAMN30626252 |
| L. Huron (13) | “ | “ | “ | “ | SAMN30626247 |
| L. Huron (14) | “ | " | “ | “ | SAMN30626248 |
| Wateree R. (1) | Big Wateree Creek, about 1 mi. ENE Wateree Creek Landing, Kershaw Co., SC | NYSM 91509 | 34.47545 | -80.891514 | SAMN30626311 |
| Wateree R. (2) | “ | “ | “ | “ | SAMN30626312 |
| Wateree R. (3) | “ | “ | “ | “ | SAMN30626313 |
| Wateree R. (4) | “ | “ | “ | “ | SAMN30626314 |
| L. Mattamuskeet (1) | Off Flying Pig Landing (NC Rt 94), Hyde Co., NC | NYSM 91508 | 35.471903 | -76.209633 | SAMN30626253 |
| L. Mattamuskeet (2) | “ | “ | “ | “ | SAMN30626254 |
| L. Mattamuskeet (3) | “ | “ | “ | “ | SAMN30626255 |
| L. Mattamuskeet (4) | “ | “ | “ | “ | SAMN30626256 |
| L. Mattamuskeet (5) | “ | “ | “ | “ | SAMN30626257 |
| L. Mattamuskeet (9) | “ | “ | “ | “ | SAMN30626258 |
| Roanoke R. (10) | Canal Gut, 3 mi. SE of Palmyra, Halifax Co., NC | NYSM 91520 | 36.038603 | -77.239103 | SAMN30626288 |
| Roanoke R. (11) | “ | “ | “ | “ | SAMN30626289 |
| Roanoke R. (12) | “ | “ | “ | “ | SAMN30626290 |
| Roanoke R. (14) | “ | “ | “ | “ | SAMN30626291 |
| Roanoke R. (15) | “ | “ | “ | “ | SAMN30626292 |
| Rappahannock R. (1) | Near Leedstown, Westmoreland Co., VA | NYSM 91507 | 38.107633 | -77.061818 | SAMN30626284 |
| Rappahannock R. (3) | “ | “ | “ | “ | SAMN30626285 |
| Rappahannock R. (4) | “ | “ | “ | “ | SAMN30626286 |
| Rappahannock R. (5) | “ | “ | “ | “ | SAMN30626287 |
| Piankatank R. (1) | Piankatank River, 2 mi. E of Glenns, Gloucester Co., VA | NYSM 91506 | 37.567101 | -76.575176 | SAMN30626278 |
| Piankatank R. (2) | “ | “ | “ | “ | SAMN30626279 |
| Piankatank R. (3) | “ | “ | “ | “ | SAMN30626280 |
| Piankatank R. (4) | “ | “ | “ | “ | SAMN30626281 |
| Piankatank R. (5) | “ | “ | “ | " | SAMN30626282 |
| Piankatank R. (6) | “ | “ | “ | “ | SAMN30626283 |
| Lake Phelps | Lake Phelps, 3 mi. SSW Creswell, Washington Co., NC | NYSM 91505 | 35.76345 | -76.469206 | SAMN30626237 |
| Schultz L. (9) | 3.5 miles southwest of Knightsville, Dorchester Co., SC | NYSM 91500 | 32.97239 | -80.27346 | SAMN30626302 |
| Schultz L. (10) | “ | “ | “ | “ | SAMN30626296 |
| Schultz L. (11) | “ | “ | “ | “ | SAMN30626297 |
| Schultz L. (15) | “ | “ | “ | “ | SAMN30626298 |
| Schultz L. (18) | “ | “ | “ | “ | SAMN30626299 |
|  |  |  |  |  |  |
| Schultz L. (19) | “ | “ | “ | “ | SAMN30626300 |
|  |  |  |  |  |  |
| Schultz L. (20) | “ | “ | “ | “ | SAMN30626301 |
|  |  |  |  |  |  |
|  |  |  |  |  |  |
|  |  |  |  |  |  |
| **Sample #** | **Locality** | **Cat. #** | **Lat.** | **Long.** | **NCBI #** |
|  |  |  |  |  |  |
| Edisto R. (1) | Edisto River, just W of Baughman’s Landing, Orangeburg Co., SC | NYSM 91501 | 33.525278 | -80.949171 | SAMN30626227 |
| Edisto R. (2) | “ | “ | “ | “ | SAMN30626228 |
| Edisto R. (3) | “ | “ | “ | “ | SAMN30626229 |
| Edisto R. (4) | “ | “ | “ | “ | SAMN30626230 |
| Edisto R. (5) | “ | “ | “ | “ | SAMN30626231 |
| Edisto R. (6) | “ | “ | “ | “ | SAMN30626232 |
| Edisto R. (7) | “ | “ | “ | “ | SAMN30626233 |
| Edisto R. (8) | Edisto River, just S of Martins landing, Orangeburg Co., SC | NYSM 91522 | 32.838217 | -80.396511 | SAMN30626234 |
| Bluff L. | James W. Webb Wildlife Center and Management Area, 4.5 mi. SW of Garnett, Hampton Co., SC | NYSM 91502 | 32.57386 | -81.31239 | SAMN30626223 |
| Wee Tee L. (14) | Wee Tee State Forest, 8.5 mi. E of St. Stephen, Williamsburg Co., SC | NYSM 91503 | 33.38581 | -79.77649 | SAMN30626315 |
| Wee Tee L. (19) | “ | “ | “ | “ | SAMN30626316 |
| Pee Dee R. (1) | Little Pee Dee River, border of Horry and Marion Counties, 11.5 mi. W of Conway, SC | NYSM 91504 | 33.83161 | -79.24972 | SAMN30626273 |
| Pee Dee R. (7) | “ | “ | “ | “ | SAMN30626277 |
| Pee Dee R. (12) | “ | “ | “ | “ | SAMN30626269 |
| Pee Dee R. (14) | “ | “ | “ | “ | SAMN30626270 |
| Pee Dee R. (17) | “ | “ | “ | “ | SAMN30626271 |
| Pee Dee R. (19) | “ | “ | “ | “ | SAMN30626272 |
| Pee Dee R. (21) | “ | “ | “ | “ | SAMN30626274 |
| Pee Dee R. (24) | “ | “ | “ | “ | SAMN30626275 |
| Pee Dee R. (26) | “ | “ | “ | “ | SAMN30626276 |
|  |  |  |  |  |  |

**Table S3:** Morphometric measurements and their definitions for Bowfins (*Amia* spp.) used herein.

| Standard Length | Anterior tip of snout to posterior end of lateral line on caudal-fin base |
| --- | --- |
| Pre-Dorsal Distance | Anterior tip of snout to anterior origin of dorsal-fin base |
| Dorsal-Fin Base Length | Anterior origin of dorsal-fin to posterior end of dorsal-fin base |
| Longest Dorsal-Fin Ray | Longest dorsal-fin ray from its base to distal tip of ray |
| Pre-Anal-Fin Distance | Anterior tip of snout to anterior origin of the anal-fin base |
| Anal-Fin Base Length | Anterior origin of anal-fin base to posterior end of anal-fin base |
| Longest Anal-Fin Ray | Longest anal-fin ray from its base to distal tip of ray |
| Anal-Fin Base to Caudal Flex Distance | Posterior end of anal-fin base to origin of caudal-fin on ventral midline |
| Pre-Pelvic-Fin Distance | Anterior tip of snout to anterior origin of pelvic-fin base |
| Pelvic-Fin Length | Anterior origin of pelvic-fin base to distal tip of pelvic fin |
| Pelvic-Fin Interspace | Width between anterior origins of left and right pelvic-fin bases |
| Pelvic Origin to Center of Anus | Anterior origin of pelvic-fin base to center of anus |
| Pelvic-Fin Origin to Pectoral-Fin Origin | Anterior origin of pelvic-fin base to anterior origin of pectoral-fin base |
| Pre-Pectoral-Fin Distance | Anterior tip of snout to anterior origin of pectoral-fin base |
| Pectoral-Fin Length | Anterior origin of pectoral-fin base to distal tip of pectoral fin |
| Body Depth at Pelvic Origin | Center point on belly between pelvic-fin origins to dorsal-fin base (measured vertically perpendicular to anterior-posterior axis of fish) |
| Pelvic-Fin Origin to Dorsal-Fin Origin | Anterior origin of pelvic-fin base to anterior origin of dorsal-fin base |
| Caudal-Peduncle Depth | Posterior end of dorsal-fin base vertically to ventral margin of peduncle, including posteriorly reflexed procurrent caudal-fin rays |
| Caudal-Peduncle Length | Posterior end anal-fin base to posterior end lateral line on caudal-fin base |
| Head Length | Anterior tip of snout to posterior fleshy margin of opercular flap |
| Head Depth | Top of head at occiput to ventral margin of head (measured vertically perpendicular to anterior-posterior axis) |
| Head Width | Maximum distance across opercula |
| Interorbital Width | Minimum distance between bony dorsal margins of left and right orbits |
| Width Between Anterior Nostrils | Width between left and right anterior nostrils (measured at centers of tube bases) |
| Width of Mouth | Width between lateral margins of dentaries (where maxilla overlaps dentary) |
| Orbit Diameter | Distance between bony rims of orbit (on anterior-posterior axis) |
| Snout Length | Anterior tip of snout to anterior rim of orbit |
| Post-Orbital Distance | Posterior orbital rim to posterior fleshy margin of opercular flap |
| Fourth Infraorbital Distance | Anterior (at orbital rim) to posterior margin of 4^th^ infraorbital (maximum distance) |
| Length of Upper Jaw | Anterior tip upper jaw (midline of pre-maxillary) to distal tip of maxilla |
| Length of Lower Jaw | Anterior tip to posterior tip of lower jaw |
| Width of Pre-Maxillary Tooth Row | Distance between lateral margins of left- and right-most teeth on premaxilla |
| Skull Length | Anterior tip of snout to posterior margin parietal bone at dorsal midline |
| Width between Posterior Nostrils | Width between left and right posterior nostrils (measured between centers of nostrils) |
| Length of Gular Plate | Anterior to posterior tip of gular plate, measured along mid-line |
| Width of Gular Plate | Maximum distance across gular plate (measured perpendicular to anterior-posterior axis of gular plate) |
| Width between Mandibles at Post. End Gular | Distance between mandibles just beyond posterior margin of gular plate |
| Posterior Nostril Diameter | Maximum distance between fleshy margins of posterior nostril (usually occurring along anterior-posterior axis) |

**Table S4:** Meristic counts and their definitions for Bowfins (*Amia* spp.) that were analyzed for this study. Additional meristic characters, including tooth and gill raker counts, are being evaluated for ongoing taxonomic analyses.

| Dorsal Fin Rays | Count of all elements (unbranched + branched) |
| --- | --- |
| Anal Fin Rays | Count of all elements (unbranched + branched) |
| Pectoral Fin Rays | Count of all elements (unbranched + branched) |
| Pelvic Fin Rays | Count of all elements (unbranched + branched) |
| Lateral Line Scales | Count of pored scales from pectoral girdle to center of caudal-fin base |
| Scales Rows Below Lateral Line | Rows from pelvic fin origin to (but not including) lateral line, counting antero-dorsally |
| Scale Rows Above Lateral Line | Rows from lateral line to dorsal fin base – counting postero-dorsally |
| Branchiostegal Rays | Total count (left + right side) |

**Table S5:** Frequency distributions of counts for selected meristic characters of Bowfin from Lake Huron (topotypes of *A. ocellicauda*) and coastal plain sites in South Carolina (including topotypes of *A. calva*), with modal values in bold. Value for lateral-line scale count of *Amia calva* holotype indicated with asterisk (*). *P*-values based on two-tailed (Wilcoxon) Mann-Whitney *U* test^5^.

|  |  |  | **Dorsal-fin Rays** | |  | |  |
| --- | --- | --- | --- | --- | --- | --- | --- |
|  | 48 | 49 | 50 | 51 | | 52 | |
| South Carolina |  | 7 | 20 | **21** | | 3 | |
| Lake Huron | **7** | 3 | 2 | 2 | |  | |
|  |  |  | *P*-same = 7.50E-05 |  | |  | |

|  |  |  |  | | **Lateral Line Scales** | | |  |  |  |  |
| --- | --- | --- | --- | --- | --- | --- | --- | --- | --- | --- | --- |
|  | 63 | 64 | 65 | 66 | | 67 | 68 | 69 | 70 | 71 | 72 |
| South Carolina |  |  |  | 2 | | 7 | 13 | **18** | 9 | 2 | 1* |
| Lake Huron | 1 | 2 | 3 | 3 | | **4** | 1 |  |  |  |  |
|  |  |  |  | *P*-same = 2.42E-07 | | | |  |  |  |  |

**Table S6:** Comparisons of selected morphometric characters between Lake Huron (topotypes of *Amia ocellicauda*) and South Carolina coastal plain populations (including likely topotypes of *Amia calva*), based on ANCOVAs with standard length as covariate^5^.

|  |  |  |  | Adjusted Regression Means | | | | | | | | | | | |  | | | |  | |  |  |  |
| --- | --- | --- | --- | --- | --- | --- | --- | --- | --- | --- | --- | --- | --- | --- | --- | --- | --- | --- | --- | --- | --- | --- | --- | --- |
| Morphometric character | |  |  | N | Lake Huron | | | N | | South Carolina | | | | | | | | | *P*-same | | | |  |  |
|  |  |  |  |  |  |  | | |  | | |  |  | | | | | | | |  |  |  |  |
| Posterior nostril diameter/interorbital width | | | | 14 | 0.076 | | 51 | | | | 0.024 | | | |  | | | 5.52E-29 | | | | | |  |
|  |  |  |  |  |  |  | | |  | | |  |  | | | | | | | |  |  |  |  |
| Dorsal-fin base/Std length | |  |  | 15 | 0.038 | | 53 | | | | 0.024 | | |  | | | 7.47E-13 | | | | | | | |
|  |  |  |  |  |  |  | | |  | | |  |  | | | | | | | |  |  |  |  |
| Pectoral-fin length/Std length | |  |  | 15 | 0.153 | | 53 | | | | 0.14 | | |  | | | 1.05E-06 | | | | | | | |
|  | |  |  |  |  |  | | |  | | |  |  | | | | | | | |  |  |  |  |
| Pelvic-fin length/Std length | |  |  | 15 | 0.128 | | 53 | | | | 0.116 | | |  | | | 2.78E-06 | | | | | | | |
|  | |  |  |  |  |  | | |  | | |  |  | | | | | | | |  |  |  |  |
| Pre-pectoral fin length/Std length | | |  | 15 | 0.263 | | 53 | | | | 0.247 | | |  | | | 1.22E-06 | | | | | | | |
|  | | |  |  |  |  | | |  | | |  |  | | | | | | | | |  |  |  |

**Supplementary References**

1. Clifford, K. M. Morphological variation in the Bowfin *(Amia calva),* with a review of nominal species: Conservation implications. (MS Thesis, SUNY College of Environmental Science and Forestry, Syracuse, NY, 2014).
2. Palumbo, J. R. (2016). Morphological diversity of Bowfins (*Amia* spp., Amiidae) among the Laurentian Great Lakes and South Carolina. (Honors Thesis, SUNY College of Environmental Science and Forestry, Syracuse, NY, 2016).
3. Sinopoli, D. A. Morphological variation of Bowfin (Amiidae: *Amia calva* Linnaeus, 1766) populations in the Mississippi River Basin: Taxonomic and conservation implications. (MS Thesis, SUNY College of Environmental Science and Forestry, Syracuse, NY, 2019).
4. Grande, L. & Bemis, W. E. A comprehensive phylogenetic study of amiid fishes (Amiidae) based on comparative skeletal anatomy. An empirical search for interconnected patterns of natural history. *J. Vertebr. Paleontol.* **18** (sup. 1), 1–696 (1998).
5. Hammer, Ø., Harper, D. A. & Ryan, P. D. PAST: Paleontological statistics software package for education and data analysis. *Palaeontol. Electron.* **4(1)**, 9 (2001). (ver. 4.10, downloaded Mar. 2022).
6. Page, L. M. & Burr, B. M. Peterson Field Guide to Freshwater Fishes of North America North of Mexico (Houghton Mifflin Harcourt, 2011).
7. Linnaeus, C. *Systema naturae sive regna tria naturae, secundum classes, ordines, genera, species, cum characteribus, differentiis, synonymis, locis*. (Vol. 1, No. 1, *Edito duodecima, reformata*, Laurentii Salvii, Holmiae [=Stockholm], 1766).
8. Richardson, J. “The Fish” in *Fauna Boreali-Americana; or the zoology of the northern parts of British America: containing descriptions of the objects of natural history collected on the late northern land expeditions, under the command of Sir John Franklin* (Part 3: i-xv + 1-327, Pls. 74-97, R. N. J. Bentley, London, 1837).
9. DeKay, J. E. *Zoology of New-York, or the New-York fauna; comprising detailed descriptions of all the animals hitherto observed within the state of New-York, with brief notices of those occasionally found near its borders, and accompanied by appropriate illustrations. Part lV. Fishes* (W. & A. White & J. Visscher, Albany, 1842).
10. Cuvier, G. & Valenciennes, A. *Histoire naturelle des poissons. Tome dix-neuvième. Suite du livre dix-neuvième. Brochets ou Lucioïdes. Livre vingtième. De quelques familles de Malacoptérygiens, intermédiaires entre les Brochets et les Clupes* (v. 19: i–xix + 1–544 + 6 pp., Pls. 554–590, C. Pitois, Paris, 1847).
11. Eschmeyer, W. N., ed. *Catalog of Fishes. Center for Biodiversity Research and Information, Special Publication 1*. Vols. 1-3: 1-2905. (California Academy of Sciences., 1998, May; also followed by on-line versions).
12. Duméril, A. H. A. *Histoire naturelle des Poissons ou Ichtyologie générale, Tome Second. Ganoides, Dipnés, Lophobranches* (Librairie encyclopédique de Roret, Paris, 1870).
